# Mg^2+^-dependent conformational changes and product release during DNA-catalyzed RNA ligation monitored by Bimane fluorescence

**DOI:** 10.1093/nar/gku1268

**Published:** 2014-12-10

**Authors:** Elisa Turriani, Claudia Höbartner, Thomas M. Jovin

**Affiliations:** 1 Scuola Normale Superiore di Pisa, Piazza dei Cavalieri 7, I-56126 Pisa, Italy; 2 Laboratory for Cellular Dynamics, Max Planck Institute for Biophysical Chemistry, 37077 Göttingen, Germany; 3 Max Planck Research Group Nucleic Acid Chemistry, Max Planck Institute for Biophysical Chemistry, 37077 Göttingen, Germany and Institute for Organic and Biomolecular Chemistry, Georg August University Göttingen, Tammannstrasse 2, 37077 Göttingen, Germany

## Abstract

Among the deoxyribozymes catalyzing the ligation of two RNA substrates, 7S11 generates a branched RNA containing a 2′,5′-linkage. We have attached the small fluorogenic probe Bimane to the triphosphate terminated RNA substrate and utilized emission intensity and anisotropy to follow structural rearrangements leading to a catalytically active complex upon addition of Mg^2+^. Bimane coupled to synthetic oligonucleotides is quenched by nearby guanines via photoinduced electron transfer. The degree of quenching is sensitive to changes in the base pairing of the residues involved and in their distances to the probe. These phenomena permit the characterization of various sequential processes in the assembly and function of 7S11: binding of Mg^2+^ to the triphosphate moiety, release of quenching of the probe by the 5′-terminal G residues of R-RNA as they engage in secondary base-pair interactions, local rearrangement into a distinct active conformation, and continuous release of the Bimane-labeled pyrophosphate during the course of reaction at 37°C. It was possible to assign equilibrium and rate constants and structural interpretations to the sequence of conformational transitions and catalysis, information useful for optimizing the design of next generation deoxyribozymes. The fluorescent signatures, thermodynamic equilibria and catalytic function of numerous mutated (base/substituted) molecules were examined.

## INTRODUCTION

Since the discovery of the first RNA-cleaving deoxyribozyme (1), several catalytic DNA molecules have been generated via *in-vitro* selection for a broad range of chemical reactions ((2–5) and references therein). Peptides and small molecules have occasionally been used as substrates, but the largest diversity of DNA-catalyzed reactions utilizes nucleic acid substrates. Besides site-specific transesterification and hydrolysis of phosphodiester backbones in DNA and RNA, DNA-catalyzed ligation reactions are of great practical interest. As an alternative to T4 ligase, deoxyribozymes can activate the 3′-terminal ribose hydroxyl group for reaction with 5′-triphosphorylated RNA leading to the formation of a covalent 3′,5′-phosphodiester bond (6,7). Another class of RNA-ligating deoxyribozymes activates internal 2′-OH groups and catalyzes the formation of 2′,5′-branched RNAs, important biochemical intermediates in RNA splicing by group II introns and the spliceosome. The chemical synthesis of 2′,5′-branched RNAs is lengthy and challenging (8–10), such that the possibility of using deoxyribozymes for RNA ligation offers particular advantages. The first representative of deoxyribozymes that form 2′,5′-branched RNA was reported by Silverman *et al.* in 2004 and was named 7S11 (11); the name refers to clone 11 of the seventh round of an *in vitro* selection experiment. Further representatives of this large class of DNA catalysts have been identified (12,13), allowing diverse combinations of DNA and RNA to be used for the synthesis of covalently branched nucleic acids (14). Applications of 2′,5′-branched nucleic acids include fundamental biochemical assays of splicing (15) and programmable manipulation of RNA folding (16,17). A closely related analog of 7S11, 10DM24 (12), serves as a versatile catalyst for site-specific labeling of RNA (18,19).

**Figure 1.**
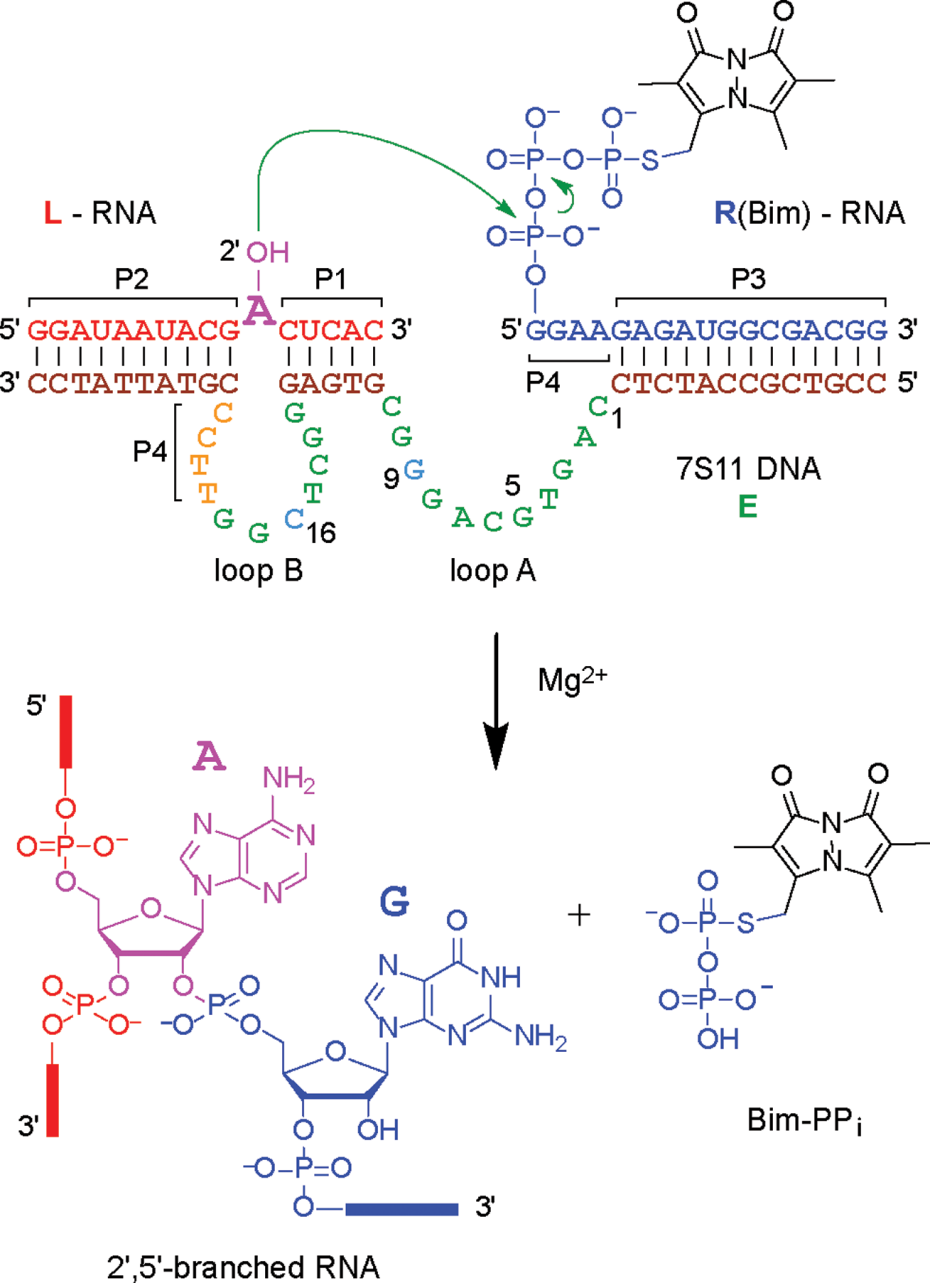
Sequence of the 7S11 deoxyribozyme (**E**) in complex with its RNA substrates (**R**, **L**) and formation of the 2′,5′-branched RNA (20,22). In the presence of a bivalent metal cation, the 2′-OH of the bulged adenosine on **L** performs a nucleophilic attack on the α-phosphate of the 5′-terminal triphosphate of **R**. In this study, the fluorescent dye Bimane (Bim) was attached at the γ-(*S*)-phosphate of **R**. The products of the reaction are a 2′,5′-branched RNA and the released pyrophosphate (PP_i_), shown here as the fluorescent Bim conjugate (Bim-PP_i_).

The 7S11 deoxyribozyme and its analogs hybridize with their RNA substrates, forming secondary (P1, P2, P3) and tertiary (P4) Watson–Crick base-paired interactions between the deoxyribozyme (**E**) and its two RNA substrates (**L**, **R**) (Figure 1), as well as two single stranded loops (A, B) in **E** (11). The active conformation of the trimolecular **L·E·R** complex is thought to be a 3-helix-junction (3HJ) (20) (Figure 1). The branching reaction proceeds through the nucleophilic attack of the 2′-OH group of a specific (unpaired) internal adenosine of **L** on the 5′-triphosphate of **R**, followed by the release of inorganic pyrophosphate. To perform its catalytic function, 7S11 requires Mg^2+^ (or Mn^2+^) as a cofactor; ligation also occurs to much lesser degree with Ni^2+^ and Co^2+^ (20). Recently, lanthanides, e.g. Tb^3+^, were used as very effective accelerating cofactors, i.e. together with Mg^2+^, in 7S11-catalyzed RNA ligation (21). The metal ions are presumed to mediate specific interactions between components of the trimolecular complex required for establishing the catalytically active structure.

Comprehensive nucleotide covariation modifications of the paired regions P1–P4 have shown that the deoxyribozyme remains active as long as the Watson–Crick base pairing in P1–P4 (Figure 1) is retained (11,20). Combinatorial mutation and nucleotide analog interference analyses have mapped the importance of each individual nucleotide and its functional groups in the catalytic loops A and B (23,24). Despite extensive information available on the influence of metal ion cofactors, pH and specific functional groups on the activity of deoxyribozymes, key structural and mechanistic features remain to be elucidated, particularly with respect to the goal of enhancing catalytic rates and product turnover.

**Figure 2.**
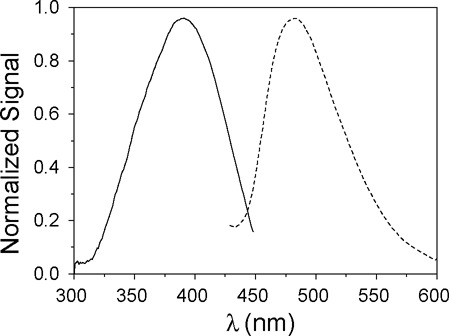
Normalized excitation (—) and emission (- - -) fluorescence spectra of the trimolecular complex LER(Bim) (2 μM). The structure of the Bimane fluorophore is shown in Figure 1. Excitation spectrum with *λ*_em_ = 480 nm and emission spectrum with *λ*_exc_ = 398 nm; pH 7.5; 15°C.

Fluorescent probes are promising tools for detailed investigation of nucleic acid folding and deoxyribozyme activation by highly sensitive fluorescence-based methods. Monobromobimane (mBBr, Bimane; Bim when coupled) is a small fluorescent compound originally developed by E.M. Kosower to label thiol groups in proteins (25–28). The small size and absence of an additional linker moiety minimizes perturbation of the molecular conformation external to the conjugation site. In addition, Bimane has very favorable fluorescent properties, such as fluorogenicity, well separated environmentally sensitive excitation and emission bands (*λ*_exc_ = 398 nm, *λ*_em_ = 480 nm; Figure 2) and high intrinsic anisotropy, leading us to explore its potential as a probe of conformational changes in polynucleotides. Indeed, this report demonstrates the great utility of Bimane in such studies. We labeled **R**-RNA selectively by synthesizing the RNA substrate in the presence of γ-*S*-GTP and exploiting the demonstrated reactivity of mBBr with thiols.

**Figure 3.**
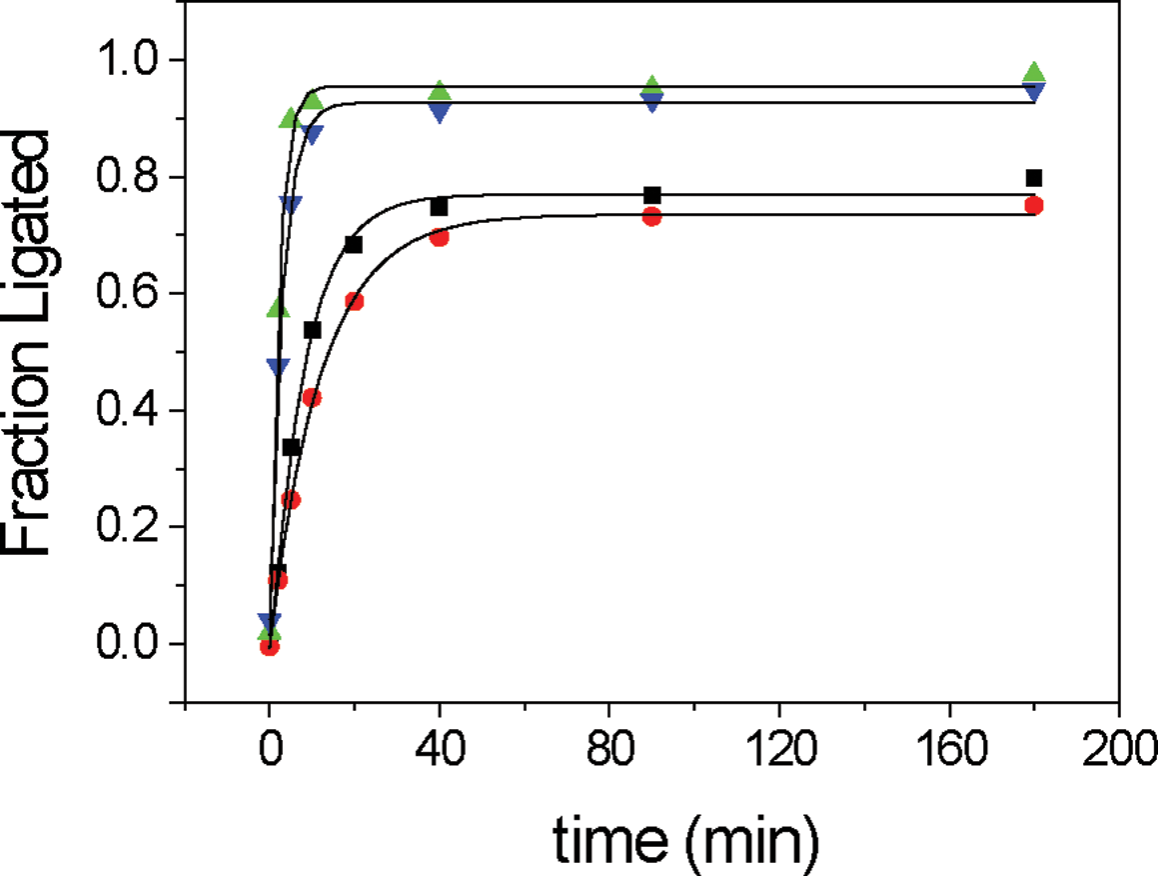
Activity assays of the various trimolecular complexes. Time courses of fractional ligation fitted with a monoexponential function. Unmodified LER (green triangles, *k*_obs_ = 0.47 ± 0.03 min^−1^), **L**(2Ap)**ER** (black squares, *k*_obs_ = 0.16 ± 0.01 min^−1^), **LER**(Bim) (blue inverted triangles, *k*_obs_ = 0.33 ± 0.02 min^−1^) and **L**(2Ap)**ER**(Bim) (red dots, *k*_obs_ = 0.083 ± 0.003 min^−1^) fluorophore labeled 7S11 substrates. Conditions: 40 mM MgCl_2_, 150 mM NaCl, pH 9; 37°C. The **L**(^32^P):**E**:**R** ratio was 1:5:10 with [**L**] = 0.1 μM.

Our experimental strategy in the study of the 7S11 deoxyribozyme incorporated the use of Förster resonance energy transfer (FRET) to monitor conformational changes during molecular assembly and function. Thus, Bimane was selected because it also fulfilled the requirements of a FRET acceptor for 2-aminopurine (2Ap) adopted as a FRET donor. 2Ap was successfully inserted in the RNA and DNA sequences, including the branch site adenosine at position 11. This substitution is compatible with the branching reaction, although the rate is somewhat reduced (29) (Figure 3). Extensive FRET determinations based on the combination of 2Ap and Bim have revealed numerous features of the Mg^2+^-dependent conformational rearrangements of 7S11 and are presented in a parallel report.

The fluorescence of Bim can be quenched via photoinduced electron transfer (PET) from tryptophan (28). This characteristic has been applied successfully to the development of the TriQ (tryptophan-induced quenching) method to map distances in proteins (30–32). Thanks to its good electron donating properties (33), guanine can act as a PET quencher in nucleic acids, similarly to tryptophan in proteins (34). For example, in fluorescein-labeled single-stranded oligonucleotides, a nearby guanine strongly quenches the probe, an effect which is abrogated upon formation of a duplex structure (35). In this work, we describe the utility of Bim in the study of the cation (Mg^2+^) dependent conformational changes in the 7S11 trimolecular complex, and as a monitor of the time course of the branching reaction. The fluorescence of Bim was also employed to study the influence of the 2Ap substitution, and of inactivating mutations of the A and B loops and of the P4 sequence of 7S11. In addition, it served to monitor the catalytic activity of 7S11 via the continuous release of Bim-PP_i_.

The addition of Mg^2+^ to the trimolecular **L·E·R** complex causes a conformational rearrangement of the strands that allows the branching reaction to take place. In accordance with established fluorescence-based methods for studying the conformational changes in ribozymes (36,37), we adopted the following model characterized by two Mg^2+^ dependent steps:

(1) \begin{equation*}
{\textbf{\textit{U}}}\rightleftharpoons{\textbf{\textit{I}}}\rightleftharpoons{\textbf{\textit{N}}}
\end{equation*}

In this scheme, ***U*** is an inactive, unfolded conformer, ***I*** is a fully paired yet unfolded conformer, i.e. an inactive intermediate, and ***N*** the final, catalytically active structure. The invocation of intermediate state ***I*** is required based on the quantitative analysis of productive reactions at 37°C as well as the bimodal steady-state titration profiles obtained at 15°C. Specific features are attributed to the two steps. In the first, the addition of the cofactor Mg^2+^ leads from ***U*** to the intermediate ***I*** conformation featuring all the paired regions P1, P2, P3 and P4 but lacking specific features of tertiary structure. In the second Mg^2+^-dependent step (${\textbf{\textit{I}}}{\rightleftharpoons}{\textbf{\textit{N}}}$), additional tertiary interactions are established and Mg^2+^ is presumed to assume an additional ‘catalytic’ role in the ligation reaction.

## MATERIALS AND METHODS

### Oligonucleotide and sample preparation

The DNA oligonucleotides and the **L**-RNA (original sequence and 2Ap labeled) substrates were prepared by solid-phase synthesis, purified by polyacrylamide gel electrophoresis (PAGE) and quantified by ultraviolet (UV) absorption. The oligonucleotide sequences are given in Figure 1 and in the Supporting Information. The **R**-RNA substrates with 5′-triphosphates and γ-*S*-5′-triphosphates were prepared by *in-vitro* transcription with T7 RNA polymerase and a synthetic DNA template. Transcription reactions were performed at 37°C for 3–5 h using 1 μM reverse strand and 1 μM promoter strand in 40 mM Tris–HCl, pH 8.0, 10 mM MgCl_2_, 10 mM dithiothreitol (DTT), 2 mM of each ribonucleoside triphosphate (NTP), and 2 mM spermidine. The reaction was quenched by addition of 50 mM ethylenediaminetetraacetic acid (EDTA) and the transcription product isolated by denaturing PAGE. The obtained oligonucleotides were quantified by UV absorption. For the experiments with ^32^P detection, radiolabeled 5′-^32^P-**L**-RNA was obtained by phosphorylation of the 5′-OH with T4 polynucleotide kinase using γ-^32^P-ATP.

Monobromobimane (mBBr) was purchased from Molecular Probes Invitrogen. The labeling of **R**-RNA with mBBr was performed using the γ-*S*-triphosphate **R**-RNA transcript (prepared by *in-vitro* transcription using γ-*S*-GTP). Small aliquots (4 × 3 μl) from a 25 mM stock solution of mBBr in acetonitrile were added to 200 μl of a 60 μM solution of γ-*S*–**R**-RNA transcript. The sample was mixed continuously during addition of the dye solution. The reaction was allowed to proceed for 4 h in the dark, under constant shaking at 25°C. Since mBBr is fluorogenic, the labeling reaction was followed by fluorescence, checking the intensity every 30 min. The labeled product was purified by denaturing PAGE and its purity was assessed by anion exchange HPLC (Supplementary Figure S1). The stability of **R**(Bim) against hydrolysis of the triphosphate was tested at 37°C (Supplementary Figure S2).

### Ligation assays

The ^32^P kinetic assays were performed following standard procedures under single-turnover conditions ((21), Supporting Information). Values of *k*_obs_ and final yield were obtained by fitting the yield-versus-time data directly to first-order kinetics.

### UV-vis spectroscopy

Spectra were recorded on a Cary 100 UV-vis spectrophotometer (Varian, Agilent Technologies, Santa Clara, USA) using Hellma (Hellma Optics, Jena, Germany) 50 and 100 μl quartz microcuvettes and temperature control. All measurements were carried out in 50 mM Na-HEPES, 150 mM NaCl, 2 mM KCl, pH 7.5. The oligonucleotide concentrations were obtained spectrophotometrically with molar extinction coefficients calculated for 260 nm according to the nearest-neighbour approximation: *ε***_L_** = 186 mM^−1^ cm^−1^, *ε***_L_**_(Ap)_ = 171 mM^−1^ cm^−1^, *ε***_E_** = 498 mM^−1^ cm^−1^, and *ε***_R_** = 207 mM^−1^ cm^−1^.

### Steady-state fluorescence

All measurements were carried out in buffered solutions 50 mM HEPES, 150 mM NaCl, 2 mM KCl, pH 7.5. Spectra were recorded on a Varian Cary Eclipse spectrofluorimeter and were corrected for lamp fluctuations (excitation) and instrumental variations (emission). The correction for the emission spectra was performed using the BAM *Spectral Fluorescence Standards* calibration kit (38).

#### Titrations

The oligonucleotides were titrated with MgCl_2_ at 15°C to minimize dissociation of the DNA:RNA trimolecular complex. Excitation (exc) and emission (em) were as follows: 2Ap, exc 306 nm and em 320–440 nm; Bim, exc 398 nm and em 410–600 nm. The measurements were performed with concentrations of the oligonucleotides in the range 0.7–7 μM. Bim fluorescence was measured with 10 nm slits in both excitation and emission monochromators.

#### Bim quantum yield

The quantum yields (*Q*_B_) of Bim in **R** (Bim) alone, in a complex with **E** (complex **ER**(Bim)) and in all the trimolecular complexes **L**(x)**E**(x)**R**(Bim), were measured using quinine sulphate (QS) as a standard (*Q*_QS_ = 0.546 (31)). Conditions: exc 398 nm, em 430–600 nm, slits exc 5 nm and em 10 nm, 15°C. Quartz cuvettes with a 1 cm pathlength were used and five spectra per measurement were averaged.

#### Steady-state anisotropy

*S*teady-state fluorescence anisotropy was recorded on the Cary Eclipse spectrofluorimeter using built-in polarizers. Steady-state anisotropy values, *r*, were determined from the parallel (*VV*) and perpendicular (*VH*) polarization signals (*V*, vertical; *H*, horizontal, the symbol order indicating excitation and emission) according to $\left\langle r \right\rangle = \left( {I_{VV} - G \cdot I_{VH} } \right)/\left( {I_{VV} + 2G \cdot I_{VH} } \right)$ where $I_{VV}$ and $I_{VH}$ are the fluorescence intensities recorded exciting the sample with vertically polarized light and recording at 90° through a vertical and horizontal polarizer, respectively. $G = I_{HV} /I_{HH}$ is a detection asymmetry correction constant determined at the appropriate excitation/emission wavelengths.

#### Fluorescence monitored kinetics of branching reaction

Equimolar concentrations (1–2 μM) of the oligonucleotides were used in 50 mM Na-HEPES, pH 7.5, 150 mM NaCl, 2 mM KCl, 40 mM MgCl_2_, 37°C. The mixtures of **L, E** and **R** were annealed by heating at 85°C for 2 min and incubating for 15 min at 25°C. The temperature was then increased to 37°C with 5 min equilibration. In some experiments an excess of **R** and **E** were employed (**L**:**E**:**R**(Bim) ratio of 10:5:1 with [**R**(Bim)] = 1 μM). A spectrum was recorded before the addition of Mg^2+^. The Bim fluorescence was recorded after the addition of Mg^2+^ at intervals ≤5 min for a minimum of 180 min. The curves were analyzed with a bi-exponential model (Equation (2))

(2) \begin{equation*}
\Delta F = a\left( {1 - e^{ - t \cdot k_{{\rm app}1} } } \right) + b\left( {1 - e^{ - t \cdot k_{{\rm app}2} } } \right)
\end{equation*}

where ${\rm \Delta }F = F_{{\rm tot}} - F_0$, *F*_0_ is the signal at *t* = 0, $k_{{\rm app}1}$ and $k_{{\rm app}2}$ are the reaction rate constants and *a* and *b* are the contributions of the two processes to the total change of signal (${\rm \Delta }F_{{\rm max}} = a + b$). The fractional contributions to the fluorescence signal of the two processes are given by ${f}_1 = a/\left( {a + b} \right)$ and ${f}_2 = b/\left( {a + b} \right)$. A more extensive analysis in terms of the thermodynamic and kinetic features of the reaction scheme represented in Equation (1) is given in Results.

### Fluorescence lifetime and time-resolved anisotropy

Measurements of the Bim fluorescence lifetimes and time-resolved anisotropy were performed with a Fluorolog3 (Horiba, JobinYvon, Glasgow, UK) spectrofluorimeter in a time-correlated single-photon counting (TCSPC) mode using the Data Station control program and Flurolog DAS6 analysis software or programs generated in *Mathematica* (see below). The excitation source was a 405 nm pulsed laser diode with a repetition rate of 1 MHz. The instrument response function (IRF) was obtained at the corresponding excitation wavelength using a 1 mg/ml sucrose scattering solution. To avoid polarization artifacts, the parallel and perpendicular polarized decays $I_{VV} (t)$ and $I_{VH} (t)$, respectively, were analyzed by a procedure designed to evaluate both decay times $\tau _i$ and rotational correlation times, $\phi _j$. These quantities were on the order of the width of the IRF (instrument response function), making it difficult to employ the routines incorporated in the DAS6 software. Instead, a multiexponential decay model (Equation (3)) was implemented in *Mathematica* (39,40). Based on statistical and graphical criteria (Supporting Information), a three-component exponential decay function was required. Its convolution with an analytical representation (41) of the IRF was used to describe the experimental decay of the total fluorescence emission at a given wavelength *λ* (in deconvoluted form, $I_{{\rm tot}} = I_{VV} + 2G \cdot I_{VH}$):

(3) \begin{equation*}
I_{{\rm tot}} = \mathop \sum \limits_{i = 1}^3 {\rm amp}_i \cdot\exp [ - t/\tau _i ]
\end{equation*}

where ${\rm amp}_i$ is the absolute amplitude (in counts) and $\tau _i$ the fluorescence lifetime of component *i*. The relative amplitude $\alpha _i$ and the amplitude-weighted mean lifetime $\tau _{{\rm amp}}$ are given by:

(4) \begin{equation*}
\alpha _i = {\rm amp}_i /\mathop \sum \limits_{i = 1}^3 {\rm amp}_i \quad {\rm and}\quad \tau _{{\rm amp}} = \mathop \sum \limits_{i = 1}^3 \alpha _i \tau _i
\end{equation*}

In the present work, $\tau _{{\rm amp}}$ was used in the evaluation of the effects of conformational changes on the fluorescence of Bim. The time-resolved anisotropy was analyzed in terms of a model for restricted rotational relaxation, with initial and limiting anisotropies ($r_0$ and $r_\infty$, respectively) and rotational correlation time $\phi$ assumed to be common to all *i* decay components. $r_{\infty }$and $\phi$ were determined by a tail-fitting procedure (Equation (5)).

(5) \begin{equation*}
r\left( t \right) = r_\infty \left( {r_0 - r_\infty } \right) \cdot {\rm exp}\left[ { - t/\phi } \right]
\end{equation*}

### Steady-state fluorescence titration

#### Titrations monitored by steady-state fluorescence intensity

The [Mg^2+^] dependence represented in titration curves was analyzed in terms of the two-step equilibrium model (Equation (1)) according to Equation (6) which defines the dependence of the relative fluorescence 〈fr〉 on the Mg^2+^-dependent equilibrium constants *K*_1_ and *K*_2_ (see below). The equilibrium constants *K*_1_ and *K*_2_ and the relative fluorescence coefficients $\varphi _{IU}$ and $\varphi _{NU}$ were obtained for all titrations.

(6) \begin{eqnarray*}
\left\langle { \rm fr } \right\rangle = \frac{ F_{\rm tot} }{ F_0 } & = & \frac{ 1 + \varphi_{IU} K_1 + \varphi _{NU} K_1 K_2 }{ 1 + K_1 + K_1 K_2 } \nonumber \\ & = & \frac{{\left[ {\textbf{\textit{U}}} \right] + \varphi _{IU} \left[ {\textbf{\textit{I}}} \right] + \varphi _{NU} \left[ {\textbf{\textit{N}}} \right]}} {{c_{{\rm tot}} = {\textbf{\textit{U}}} + {\textbf{\textit{I}}} + {\textbf{\textit{N}}}}} \
\end{eqnarray*}

The quantity $F_{{\rm tot}}$ is the total fluorescence and the equilibrium constants $K_1$ and $K_2$ are defined, respectively, as $K_1 = [{\textbf{\textit{I}}}]/[{\textbf{\textit{U}}}] = K_{1{\rm app}} \left[ {{\rm Mg}^{{\rm 2 + }} } \right]$ and $K_2 = [{\textbf{\textit{N}}}]/[{\textbf{\textit{I}}}] = K_{2{\rm app}} \left[ {{\rm Mg}^{{\rm 2 + }} } \right]$, implying a first-order dependence on the cation concentration such that $F_0 = F_{{\rm tot}}$ in the absence of Mg^2+^. The total concentration of the trimolecular complex is given by $c_{{\rm tot}} = \left[{\textbf{\textit{U}}} \right]\left( {1 + K_1 \left( {1 + K_2 } \right)} \right)$. The relative fluorescence coefficients $\varphi _{IU}$ and $\varphi _{NU}$ are defined as the ratios $\left( {\varphi _I /\varphi _U } \right)$ and $\left( {\varphi _N /\varphi _U } \right)$, where $\varphi _i$ is a brightness parameter proportional to the product of the quantum yield $Q_i$ and the extinction coefficient $\varepsilon _i$ of species *i.* All measurements were performed in the presence of a large excess of MgCl_2_ over nucleic acid such that $\left [{{\rm Mg}^{{2 + }}} \right]_{{\rm free}} \approx \left[{{\rm Mg}^{{2 + }}}\right]_{{\rm tot}}$. In the titration of the complex containing the inactive **E**(G9A) mutant of the deoxyribozyme the data were fit with a single conformational change (with one equilibrium constant and corresponding change in fluorescence).

#### Titrations monitored by steady-state anisotropy

To evaluate the anisotropy of Bim in the ***U***, ***I*** and ***N*** conformations (Figure 1) of the trimolecular complex **L**(dAp)**ER**(Bim), the data were treated according to the following method. The measured (mean) steady-state anisotropy *r* is given by the intensity-weighted average of the individual anisotropies $r = f_U r_U + f_I r_I + f_N r_N$, where $f_U$, $f_I$, and $f_N$, respectively, are the fractional intensities of Bim fluorescence in each conformation and $r_U$, $r_I$ and $r_N$ are the corresponding anisotropies. After substitutions in Equation (6) by the defined quantities, we obtain

(7) \begin{equation*}
r = \frac{{r_U + r_I \varphi _{IU} K_1 + r_N \varphi _{NU} K_1 K_2 }}{{1 + \varphi _{IU} K_1 + \varphi _{NU} K_1 K_2 }}
\end{equation*}

where $\varphi _{IU}$ and $\varphi _{NU}$ are the relative fluorescence coefficients defined previously for the species ***I*** and ***N***, relative to the fluorescence coefficient of the species ***U***. The values of $K_1$ and $K_2$, as well as those of $\varphi _{IU}$ and $\varphi _{NU}$, obtained from the steady state-fluorescence titrations were used in the analyses.

## RESULTS

### Assessment of the influence of Bim on the kinetics of the branching reaction

In order to draw significant conclusions from the fluorescence study of the folding of 7S11 trimolecular complex in the presence of the metal cofactor Mg^2+^, the impact of labeling with Bimane and 2-aminopurine on the catalytic activity was evaluated. The activity of 7S11 complex is retained when using a 5′-adenylated substrate, in which the pyrophosphate leaving group is replaced with AMP as a leaving group (22). This suggested that the labeling of the 5′-triphosphate with a fluorescent probe could be performed without loss of activity. The branching reaction was performed with a 5′-^32^P on the **L**-RNA substrate; aliquots were withdrawn at specific time points, quenched to stop the reaction, and analyzed by gel electrophoresis (11,20,22,43). The activity of the enzyme in the presence of **L**(Ap) and **R**(Bim) labeled substrates was determined under the conditions for the branching reaction: 40 mM Mg^2+^, 37°C, pH 9. Four types of kinetic assays were performed: in the absence of the probes, and in the presence of 2Ap only, Bim only or both (Figure 3).

The activity assays revealed that the reaction rate was mostly affected by the substitution of the branch site adenosine with the 2Ap (3-fold reduction in the reaction rate), while in the presence of Bim on the γ-phosphate on the 5′-end of the **R**-RNA substrate, the reaction rate was only ∼30% slower. These control experiments demonstrated that the catalytic activity and active conformation of the system are substantially retained in the presence of the fluorophores.

### Equilibrium folding monitored by steady-state fluorescence of the original 7S11 sequence

**Figure 4.**
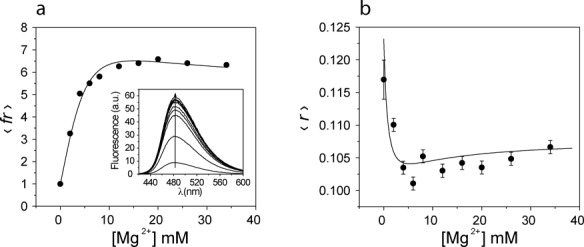
Fluorescence titration of the **L**(dAp)**ER**(Bim) trimolecular complex with MgCl_2_ followed by steady state fluorescence emission (**a**) and anisotropy (b) upon direct excitation of Bim. (a) Changes in 〈fr〉. Inset: fluorescence emission spectra of Bim during the titration. The titration curve was recorded with *λ*_exc_ = 398 nm and *λ*_em_ = 480 nm. (**b**) Titration recording the steady state anisotropy of Bim; *λ*_exc_ = 398 nm, *λ*_em_ = 450–560 nm. The titration was analyzed with the two-step equilibrium model (Equation (6)), obtaining the same equilibrium constants from both the fluorescence emission and the steady state anisotropy. Conditions: 1 μM **L**(dAp)**ER**(Bim), 0–40 mM MgCl_2_, 150 mM NaCl, 2 mM KCl, 40 mM Na-HEPES, pH 7.5; 15°C.

Three different trimolecular complexes containing the original 7S11 sequence were titrated with MgCl_2_: **LER**(Bim), containing the unmodified **L**-RNA substrate; **L**(Ap)**ER**(Bim), in which the branch site nucleotide was substituted with 2Ap; and **L**(dAp)**ER**(Bim), in which the branch site nucleotide was substituted with 2′-deoxy-2Ap. The addition of MgCl_2_ caused an increase in 〈fr〉 (relative fluorescence of Bim) in all combinations (Figure 4a and Supplementary Figure S3). In the case of **LER**(Bim), the increase was 7-fold (Supplementary Figure S3a). No shift in either the excitation or emission spectra was observed; an example of the emission spectra is shown in the inset of Figure 4a.

With **L**(Ap)**ER**(Bim) sample, the increase in 〈fr〉 was 4-fold (Supplementary Figure S3b). As shown in the previous section, the substitution of 2Ap on the branch site nucleotide of the **L**-RNA does not inactivate the system. **L**(dAp)-RNA on the other hand lacked the 2′-OH group that acts as a nucleophile during the branching reaction such that **L**(dAp)**ER**(Bim) was inactive. In this sample, the value of 〈fr〉 increased 6.5-fold upon titration with MgCl_2_ (Figure 4a).

For the samples containing **E**-DNA (original 7S11 sequence), the titration curves were analyzed together in a global fit with the two-step model described by Equations (1) and (6), yielding the apparent equilibrium constants ^E^*K*_1app_ = 58 ± 12 M^−1^ and ^E^*K*_2app_ = 400 ± 130 M^−1^ (the prefix E in ^E^*K*_1app_ and ^E^*K*_2app_ indicates samples containing **E**-DNA) (Supplementary Figure S3, panels (a) and (b)). The samples yielded different relative fluorescence coefficients, allowing the calculation of the quantum yields of Bim in the ***U***, ***I*** and ***N*** conformations in the three trimolecular complexes (Table 1). Among the samples containing the **E**-DNA sequence, only in **L**(dAp)**ER**(Bim) was the quantum yield of Bim in the species ***I*** higher than in the species ***N*** (Table 1). The value of 〈fr〉 increased 6-fold, reaching its maximum at 10 mM Mg^2+^ and decreasing at higher concentrations.

**Table 1.** Quantum yields of Bim in the *U*, *I* and *N* conformations in the different trimolecular complexes

Complex	10^2^*Q_U_*	10^2^*Q_I_*	10^2^*Q_N_*
**LER**(Bim)	0.54 ± 0.01	4.0 ± 0.1	4.0 ± 0.1
**L**(Ap)**ER**(Bim)	0.67 ± 0.01	2.8 ± 0.1	2.8 ± 0.1
**L**(dAp)**ER**(Bim)	0.70 ± 0.01	13 ± 2	3.9 ± 0.1
**L**(Ap)**E**(G9A)**R**(Bim)	0.73 ± 0.01	2.20 ± 0.04	
**L**(Ap)**E**(C16G)**R**(Bim)	0.70 ± 0.02	2.3 ± 0.1	1.4 ± 0.2
**LE**(P4)**R**(Bim)	0.68 ± 0.01	1.45 ± 0.03	0.96 ± 0.03
**L**(Ap)**E**(P4)**R**(Bim)	0.64 ± 0.01	1.49 ± 0.04	0.97± 0.03

Values obtained from the analysis of the data with Equation ((6)). Conditions: 1 μM **L**(X)**E**(Y)**R**(Bim), 0–40 mM MgCl_2_, 150 mM NaCl, 2 mM KCl, 40 mM Na-HEPES, pH 7.5; 15°C; *λ*_exc_ = 398 nm, *λ*_em_ = 480 nm.

The steady state emission anisotropy of Bimane elicited by direct excitation was recorded while titrating **L**(dAp)**ER**(Bim) with MgCl_2_ (Figure 4b). The **L**(dAp)-RNA chimeric substrate was chosen to ensure that the branching reaction did not take place. In the absence of Mg^2+^ the steady-state anisotropy of the dye was low, as expected for a fluorophore attached to a mobile linker such as the triphosphate. The value of *r* showed a biphasic trend upon addition of MgCl_2_, first decreasing (reaching a minimum at 8 mM Mg^2+^) and then increasing slightly at higher concentrations (Figure 4b). The data were analyzed with Equation (7), yielding steady-state anisotropy values of Bimane in the different conformations: $r_U$ = 0.123 ± 0.008, $r_I$ = 0.098 ± 0.004 and $r_N$ = 0.108 ± 0.002.

### Equilibrium folding monitored by steady-state fluorescence of inactive deoxyribozyme mutants

The mutation of specific nucleotides on the catalytic loops A and B causes the inactivation of the deoxyribozyme (20,23,24), without affecting the base pairing of the P1–P4 regions. In order to investigate the effects of the mutations on the structure and folding of the trimolecular complexes, **L**(Ap)**E**(G9A)**R**(Bim) (mutated in loop A) and **L**(Ap)**E**(C16G)**R**(Bim) (mutated in loop B) were titrated with MgCl_2_. In the **E**(G9A) and **E**(C16G) containing complexes, the increase in fluorescence intensity of Bim was reduced. The addition of Mg^2+^ (0–40 mM) to **L**(Ap)**E**(G9A)**R**(Bim) and **L**(Ap)**E**(C16G)**R**(Bim) caused an increase in the fluorescence intensity of only 3.0-fold and 2.4-fold, respectively. In the case of **L**(Ap)**E**(G9A)**R**(Bim) a single increase in fluorescence was observed (Supplementary Figure S3c) and a single step model was used to analyze the titration data, yielding the equilibrium constant ^E^^(G9A)^*K*_1app_ = 350 ± 20 M^−1^. In **L**(Ap)**E**(C16G)**R**(Bim) on the other hand, the fluorescence showed a biphasic behavior (Supplementary Figure S3d) and the data were analyzed with the two-step equilibrium model described in Equations (1) and (6). The obtained equilibrium constants were: ^E(C16G)^*K*_1app_ = 490 ± 100 M^−1^ and ^E^^(C16G)^*K*_2app_ = 43 ± 32 M^−1^. The quantum yields from the analysis of the titration curves are reported in Table 1.

The **E**(P4) DNA sequence is characterized by the mutation of all four nucleotides of the P4 region (TTCC to AAGG) in **E**-DNA. The formation of the P4 helix is thereby impaired, such that the complex cannot assume the active 3-helix junction conformation and is inactive (20). For the trimolecular complexes containing the P4 mutant **E**(P4), **LE**(P4)**R**(Bim) and **L**(Ap)**E**(P4)**R**(Bim), the increase in Bim fluorescence in the 0–40 mM [Mg^2+^] range was limited to 1.5-fold (Supplementary Figure S3e and f). The Bim fluorescence data from the titration of **LE**(P4)**R**(Bim) and **L**(Ap)**E**(P4)**R**(Bim) were analyzed together in a global fit with the model described in Equations (1) and (6). The obtained equilibrium constants were ^E(P4)^*K*_1app_ = 590 ± 50 M^−1^ and ^E(P4)^*K*_2app_ = 52 ± 6 M^−1^.

### Equilibrium folding monitored by time resolved fluorescence

**Figure 5.**
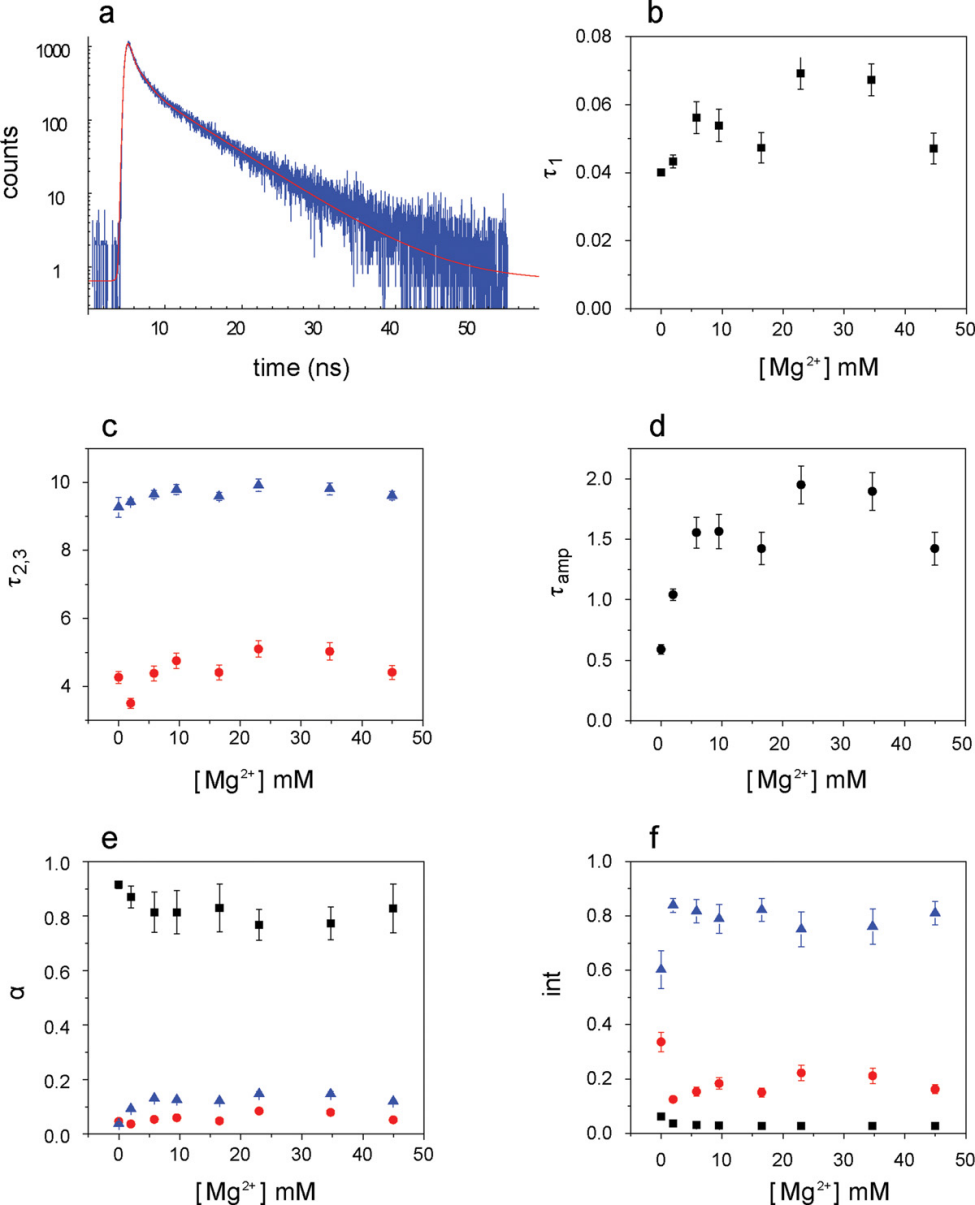
Time-resolved fluorescence decay of Bimane. (**a**) Decay curve in the trimolecular complex **L**(dAp)**ER**(Bim) at 45 mM [Mg^2+^]. The decay curve was fit with three lifetime components; (**b**–**d**) [Mg^2+^] dependence of the single fluorescence lifetime components and of the amplitude averaged fluorescence lifetime (black squares, $\tau _{1}$, red circles, $\tau _{2}$, blue triangles, $\tau _{3}$ and black circles, $\tau _{{\rm amp}}$); (**e**) fractional amplitudes *α*; and (**f**) fractional intensities ($\alpha _i \cdot \tau _i$) of the three decay components; Conditions: 4.2 μM **L**(dAp), 4.1 μM **E**, 4.0 μM **R**(Bim), pH 7.5; 15°C; *λ*_exc_ = 405 nm, *λ*_em_ = 480 nm.

The time-resolved fluorescence emission for Bim in the presence of increasing concentrations of MgCl_2_ was monitored. Upon analysis of the decay curves with different models, the comparison of the residuals led to the selection of a three-exponential decay model (Figure 5a) as necessary and sufficient (Supplementary Figure S4 and Supplementary Table S1). The **L**(dAp) substrate was selected to inhibit the branching reaction during the measurement. The presence of 2Ap did not affect the fluorescence of Bim. The addition of Mg^2+^ to **L**(dAp)**ER**(Bim) led to an increase in the fluorescence lifetime of Bim compared to the sample lacking Mg^2+^. Upon addition of MgCl_2_ to the tri-molecular complex, the shortest lifetime (*τ*_1_) increased, whereas *τ*_2_ and *τ*_3_ were unaltered (Figure 5b,c and d). The samples also showed significant changes in relative amplitudes, with a 20% decrease in α_1_ and a 3-fold increase of α_3_, the relative amplitude corresponding to the longest lifetime (Figure 5e). Both factors contributed to the increase of the amplitude averaged lifetime *τ*_amp_ (Figure 5d, Supplementary Table S2). These changes were also reflected in the corresponding intensities (Figure 5f).

**Figure 6.**
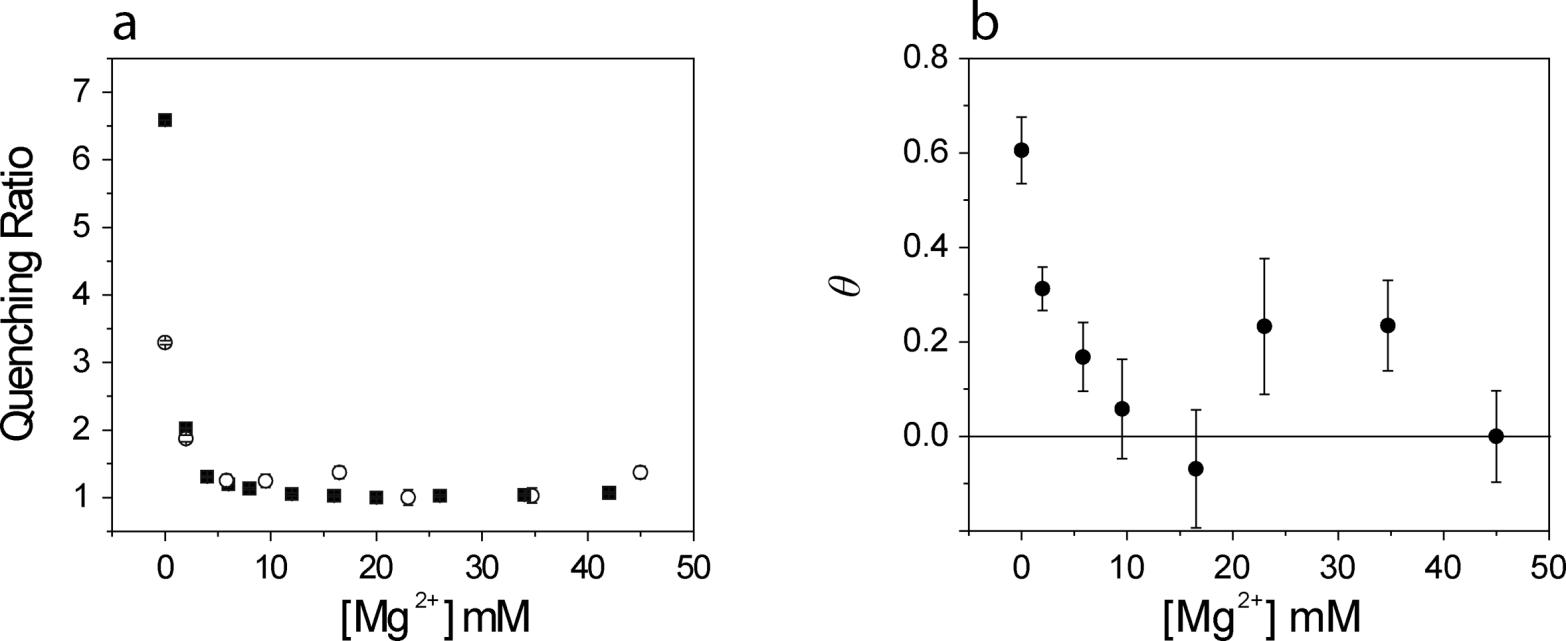
Fluorescence intensity and lifetime ratio functions (see text) for **L**(dAp)**ER**(Bim) a function of [Mg^2+^]. (**a**) $F_{{\rm max}} /F_k$, (open circles) and $\tau _{{\rm ampmax}/} \tau _{{\rm amp},k}$ (filled squares). (**b**) Fraction of probe (*θ*) involved in static G–Bim complex formation.

The changes in both steady-state and time-resolved fluorescence upon addition of Mg^2+^ to the Bim labeled trimolecular complexes suggested that the quenching by guanine could be both dynamic and static (involving the formation of ground state complexes). To assess this possibility, the steady-state fluorescence and time-resolved fluorescence data were processed in a manner similar to the TrIQ approach of Mansoor *et al.* (30). Two ratios were calculated. In $F_{{\rm max}} /F_k$, $F_{{\rm max}}$ is the fluorescence intensity of Bim in **L**(dAp)**ER**(Bim) at 40 mM Mg^2+^, and $F_k$ is the fluorescence intensity at a particular [Mg^2+^]*_k_*; these values were calculated using the quantum yields and the equilibrium constants obtained from steady state fluorescence titrations. In the second ratio $\frac{{\tau _{{\rm ampmax}} }}{{\tau _{{\rm amp},k} }}$, $\tau _{{\rm amp}}$ was used because in the presence of multi-component decays, its value is directly proportional to the steady state fluorescence intensity in the absence of static quenching. The Mg^2+^ dependence of the two ratios is given in Figure 6a. The discrepancy at lower concentrations is indicative of the presence of non-fluorescent guanine (G)–Bim complexes. The fraction of dyes involved in static (non-fluorescent) complexes, *θ*, can be calculated according to equation (8)

(8) \begin{equation*}
\theta = 1 - \frac{{{\bf \tau }_{{\rm ampmax}} }}{{{\bf \tau }_{{\rm amp},k} }} \cdot \frac{{F_k }}{{F_{{\rm max}} }}
\end{equation*}

Figure 6b demonstrates that *θ* was significantly > 0 for [Mg^2+^] ≤ 10 mM, indicating that a considerable fraction of Bim molecules were involved in static complexes and thus completely quenched. At [Mg^2+^] ≥ 10 mM, *θ* ≈ 0, from which we conclude that quenching of the corresponding molecular species was quantitatively dynamic. The structural interpretation of this result is given in the ‘Discussion’ section.

**Figure 7.**
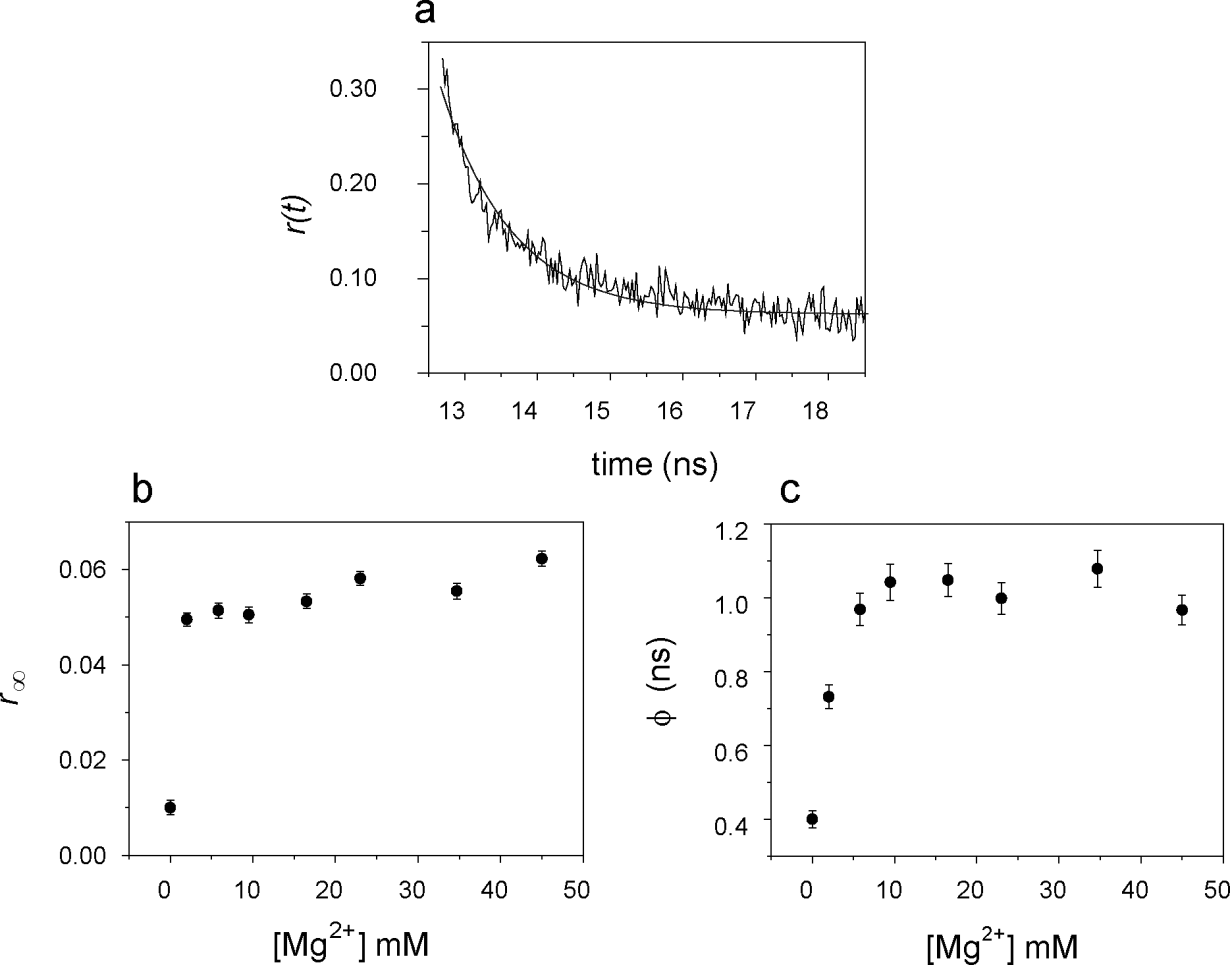
Time dependent anisotropy of Bim: dependence on the concentration of Mg^2+^. (**a**) Anisotropy decay of Bim in the **L**(dAp)**ER**(Bim) trimolecular complex at 45 mM MgCl_2_, decay curve analyzed with Equation (5); (**b**) residual anisotropy $r_\infty$; (**c**) rotational correlation time *ϕ*. Conditions: 4.2 μM **L**(dAp), 4.1 μM **E**, 4.0 μM **R**(Bim), pH 7.5; 15°C; *λ*_exc_ = 405 nm, *λ*_em_ = 480 nm.

The [Mg^2+^] dependence of the time-resolved anisotropy of Bimane was also recorded. The curves were well fit by a mono-exponential decay model (tail fitting, Figure 7a). In the absence of Mg^2+^ the limiting anisotropy $r_\infty$ was close to zero. The value of $r_\infty$ increased abruptly (by 0.04 units) with the first addition of MgCl_2_, with a further smaller increase at higher concentrations (Figure 7b). The rotational correlation time (*ϕ*) also increased upon addition of MgCl_2_ (Figure 7c), following the same trend shown by the amplitude averaged fluorescence lifetime (Figure 5d).

### Branching reaction kinetics monitored by Bimane fluorescence

**Figure 8.**
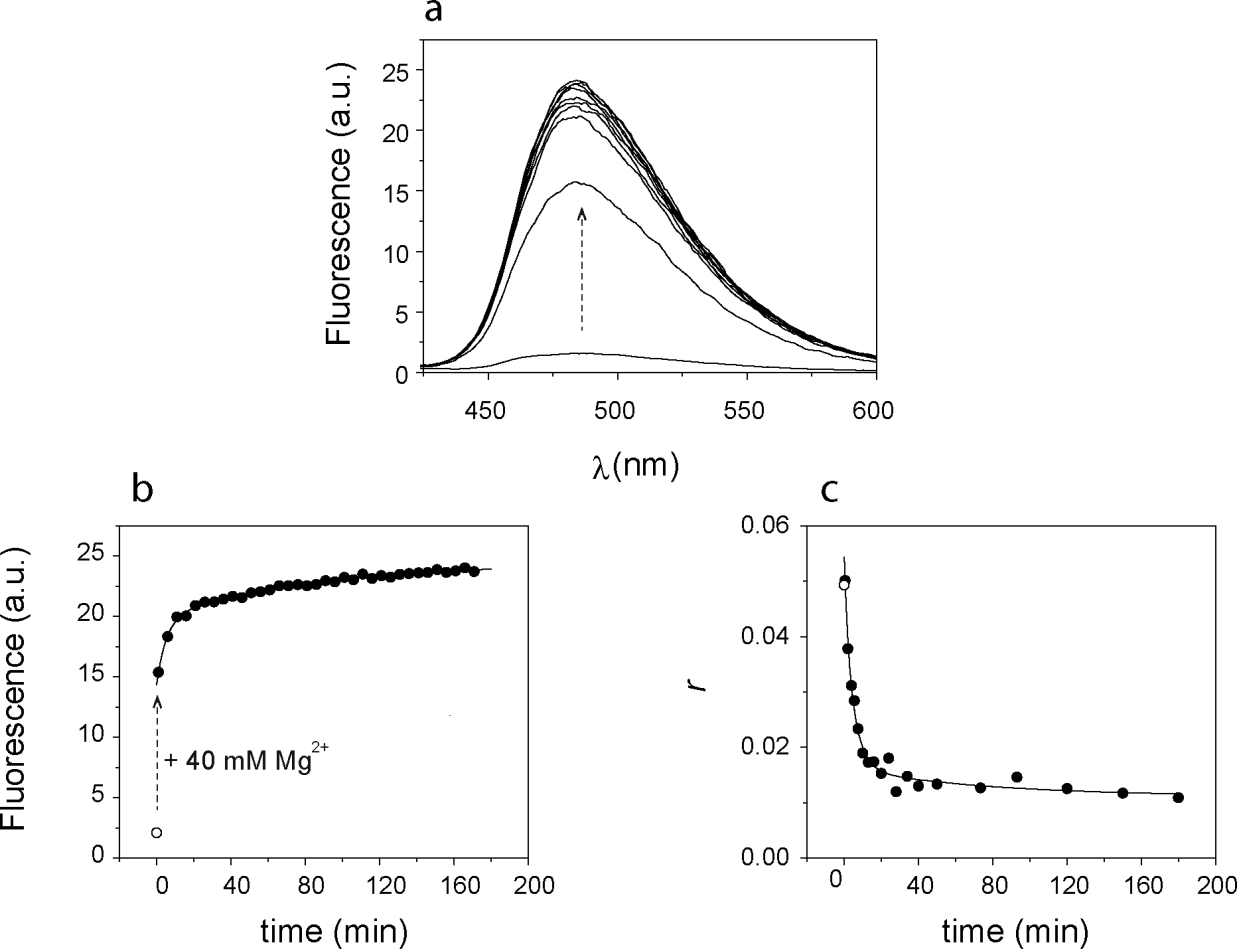
Kinetics of branching reaction with Bim-labeled **R**(Bim)-RNA after addition of 40 mM Mg^2+^. (**a**) Emission spectra as a function of time; (**b**) time course followed by fluorescence intensity and (**c**) steady-state anisotropy. The dashed arrow in panels (a) and (b) indicates the change in intensity upon addition of 40 mM Mg^2+^; the steady state fluorescence (b) and anisotropy (c) of **LER**(bim) in the absence of Mg^2+^ is indicated by an empty circle. Conditions: 2 μM **LER**(Bim), 40 mM [Mg^2+^], pH 7.5; 37°C; *λ*_exc_ = 398 nm, *λ*_em_ = 470–520 nm. See text for description of fits.

The addition of Mg^2+^ at 37°C and pH 7.5 to a preformed, active **LER**(Bim) complex led to a large increase in fluorescence (Figure 8a) characterized by three steps: (i) ‘instantaneous’ (<1 min) ∼5-fold increment, (ii) a ∼2.5-fold increase in the 10 min range and (iii) a slower (∼100 min) increase of similar magnitude (Figure 8b). The emission spectrum was unaltered and thus the change is attributable to differing quantum yields of the conformational states and reaction products involved. An enhancement of Bimane fluorescence has also been described during the cleavage of Bim-labeled peptides containing tryptophan (28). The kinetic curves were fit well with a two-exponential model (Equation (2)). For the reaction with **LER**(Bim) depicted in Figure 8a, the kinetic parameters were *k*_obs1_ = 0.19 ± 0.02 min^−1^ and *k*_obs2_ = 0.011 ± 0.002 min^−1^, with corresponding *f*_1_ = 0.48 ± 0.02 and *f*_2_ = 0.51 ± 0.02. The progress of the reaction was also followed by monitoring the steady-state anisotropy of Bim, which decreased dramatically during steps (ii) and (iii), but not (i) of the reaction (Figure 8c). The kinetic constants (*k*_obs1_ = 0.17 ± 0.01 min^−1^, *k*_obs2_ = 0.008 ± 0.002 min^−1^) from the anisotropy time trace were comparable to those obtained from the fluorescence intensity. With the **L**(Ap) substrate, the reaction rate derived from analysis of the Bim fluorescence was 75% slower (*k*_obs1_ = 0.046 ± 0.005 min^−1^, *k*_obs2_ = 0.011 ± 0.001 min^−1^, fractional contributions *f*_1_ = 0.31 ± 0.05 and *f*_2_ = 0.69 ± 0.11) (Supplementary Figure S5).

The *k*_obs_ were invariant (±20%) for equimolar trimolecular constructs or those formed with a 10:5:1 **L**:**E**:**R**(Bim) stoichiometry; nor did they vary significantly with the absolute concentration of **R**(Bim) over a two-fold range (1–2 μM). This feature and the nature of the three-phase kinetic progression indicate that the overall reaction involves a sequence of first-order steps initiated by the addition of Mg^2+^ and represented in Equation (9).

(9) \begin{equation*}
\begin{array}
{*{20}l} {step\quad\,\,\, i\quad\,\, ii\quad \,\, iii} \\ {\textbf{\textit{\qquad U}}\mathop\rightleftharpoons\limits_{K_{{\rm d}1} } \textbf{\textit{I}}\mathop\rightleftharpoons\limits_{k_{ - 2} }^{k_2 } \textbf{\textit{N}}\mathop \to \limits^{k_{cat} } \textbf{\textit{P}}_{1,2} } \\
\end{array}
\end{equation*}

In this scheme, step (i) is attributed to the rapid establishment of an equilibrium distribution between ***U*** and ***I***, step (ii) to the slower equilibration between ***I*** and the active state ***N***, and step (iii) to the catalytic formation of the branched RNA (product *P*_1_) and the concerted release of Bim-PP_i_, (product *P*_2_) into the medium. The analytical solution to Equation (9) leads to a biexponential process corresponding to *k*_obs1_ and *k*_obs2_

(10) \begin{eqnarray*}
k_{\rm obs1}+k_{\rm obs2} &=& \frac{k_2 }{1 + K_{{\rm d}1} } +k_{ - 2}+k_{\rm cat};\nonumber\\ k_{\rm obs1} \cdot k_{\rm obs2} &=& \frac{k_{2}k_{\rm cat}}{1+K_{\rm d1}};\nonumber\\ K_{\rm d2} &=& \frac{k_{-2}}{k_2 }
\end{eqnarray*}

as well as to expressions for the amplitudes and thereby the time dependent concentrations of each component and the corresponding fluorescence intensities (Equation (6)). The steady-state anisotropy would have the following form (Equation (11)) derived as an extension of Equation (7)

(11) \begin{eqnarray*}
&& { \left\langle {r(t)} \right\rangle = } \\ &&{ \frac{\displaystyle{r_U \left[ {U(t)} \right] + r_I \varphi _{IU} \left[ {I(t)} \right] + r_N \varphi _{NU} \left[ {N(t)} \right] + r_{P_2 } \varphi _{P_2 U} \left[ {P_2 (t)} \right]}}{{\displaystyle\left[ {U(t)} \right] + \varphi _{IU} \left[ {I\left( t \right)} \right] + \varphi _{NU} \left[ {N\left( t \right)} \right] + \varphi _{P_2 U} \left[ {P_2 \left( t \right)} \right]}} } \nonumber
\end{eqnarray*}

The analysis of the data of Figure 8 and of other related experiments in 40 mM Mg^2+^ at 37°C yielded the following values for the parameters valid for **LER**(Bim) reaction in 40 mM Mg^2+^ at 37°C:

\begin{equation*}
\begin{array}
{*{20}l} {\varphi _{IU} ,8;\varphi _{NU} ,10;\varphi _{P_2 U} ,12;} \\ {K_{{\rm d1}} ,0.2;k_2 ,0.2\,{\rm min}^{ - 1} ;k_{ - 2} ,0.02; K_{{\rm d2}} ,0.1; k_{{\rm cat}} ,0.01\,{\rm min}^{ - 1} ;} \\ { r_U ,0.05;r_I ,0.06;r_N ,0.01;r_{P_2 } ,0.01.} \\
\end{array}
\end{equation*}

The standard errors given by the fits (using *Mathematica* Nonlinear Model Fit) were very low; analyses of numerous data sets alone or in global combination yielded parameter values within ∼30% of those cited here. The interpretation of these results is given in the Discussion section.

## DISCUSSION

### Conformational information obtained from steady state fluorescence

This study of deoxyribozyme 7S11 has demonstrated that the fluorescence of Bimane constitutes a valuable, versatile probe of structure and function of synthetic nucleic acids with catalytic capabilities. Bim was attached to the triphosphate 5′ terminus of the **R**-RNA. The changes in steady-state and time-resolved fluorescent parameters—quantum yield, lifetime, anisotropy—reflect changes in the immediate microenvironment of the probe as well as in global features of the trimolecular complex, for example, the formation of key tertiary base pairing interactions (P4 region). The latter depend on multiple interactions of the essential cofactor Mg^2+^ mediating the concentration-dependent transition between an inactive ***U*** conformation and the catalytic ***N*** end state, passing via an intermediate inactive conformation ***I***.

In proteins, Bim fluorescence is quenched by tryptophan and tyrosine side chain (26–28,30–32) via PET; this effect is distance dependent. In nucleic acids, guanine can act as a quencher by virtue of its low oxidation potential compared to the other nucleobases (33,35). The Bim-triphosphate (negatively charged) 5′-end of the **R**-RNA substrate is in close proximity to two guanine residues. The increase in the fluorescence emission intensity of Bim in the various 7S11 based constructs upon titration with Mg^2+^ is thus attributable to pairing of the neighboring Gs with the corresponding Cs in the P4 region of the deoxyribozyme, as well as on complexation of Mg^2+^ and the 5′-triphosphate, which alone leads to a two fold increase in fluorescence in the single stranded **R**(Bim) (Supplementary Figure S2). Both the inactive **L**(dAp)**ER**(Bim) and the active **L**(Ap)**ER**(Bim) manifested this enhancement of fluorescence, suggesting that the absence of the OH group in the branch site 2Ap does not inhibit the pairing of the P4 nucleotides. The comparison of the **LER**(Bim) results with those obtained for the trimolecular complexes containing the inactive mutants **E**(G9A) and **E**(C16G) suggests that Mg^2+^ can trigger the formation of the P4 paired region in these inactive hybrids. The titration curve obtained for the **L**(Ap)**E**(G9A)**R**(Bim) was analyzed with a model involving a single conformational change. The mutation of G9 on loop A disrupts a series of three guanines that are essential for activity (23,24) and might be involved in tertiary interactions. The hypothesis that this mutation inhibits the obtainment of the active structure ***N*** was formulated considering the single trend in fluorescence observed in Bim-fluorescence (and the 2Ap-fluorescence data and the results of MD simulations shown in a separate report). The smaller signal change with **E**(P4), in which the two neighboring G's are incapable of forming base pairs, arises exclusively from direct complexation of Mg^2+^ with the triphosphate moiety.

The similar quantum yields (*Q**_U_***) of Bim in the ***U*** forms of the studied constructs indicates that the microenvironment of the probe was invariant in the absence of Mg^2+^, supporting the conclusion that the 5′- end bases of the **R**-RNA substrate (overhanging bases of P4) are mostly unpaired in the ***U*** conformer. The accompanying biphasic changes in the steady-state anisotropy of Bim upon addition of Mg^2+^ corresponded well to the changes observed in intensity, and are attributable to a relative increase of the rotational correlation time smaller than the corresponding change in fluorescence lifetime, as represented in the general relationship $r^{ - 1} \propto (1 + \tau /\phi)$ (see next section).

### Conformational information obtained from time-resolved fluorescence

To further explore the nature of the quenching mechanism, the fluorescence decay of Bim in the trimolecular complex **L**(dAp)**ER**(Bim) was measured at various concentrations of Mg^2+^. A three-component analysis was required in order to fit the data. The three lifetimes as well as their amplitude weighted mean, $\tau _{{\rm amp}} ,$ increased with the Mg^2+^ concentration, in the latter case ∼3-fold by 10 mM. We interpret this result in terms of the TriQ method introduced by Mansoor et al. (30–32) in which the quenching of Bimane fluorescence by PET from tryptophan is used to map distances in proteins using time-resolved fluorescence spectroscopy. Quenching by PET occurs over short distances (5–15 Å) and thus the method can be considered complementary to FRET, which is generally applied to the study of larger distances. The combined analysis of the dequenching of Bim based on both the steady-state intensity and excited-state lifetime(s) data indicates that the transition from ***U*** to ***I*** involves the loss of largely static intra-chain complexes of Bim with one or more G residues to a largely dynamic, less effective mode of quenching as the P4 helix forms. The transition from ***I*** to ***N*** appears to lead to still further dequenching, reflecting structural readjustments in the local vicinity of the probe.

As stated above, in the trimolecular complex containing the **R**(Bim)-RNA substrate, the addition of Mg^2+^ does not lead to an increase in the distance between the probe and the guanine on the 5′-end of the **R**(Bim) substrate. Instead, Mg^2+^ induces the pairing of the P4 region and also forms a complex with the triphosphate on **R**-RNA 5′-end, the latter occurring at low (3 mM) Mg^2+^ concentration. The changes observed in the steady state fluorescence and fluorescence lifetime of Bim in **L**(dAp)**ER**(Bim) confirm that the two P4 guanines lose their ability to quench Bim upon base pairing with the corresponding cytosines. A similar finding, i.e. a decrease in the PET-induced quenching of fluorescein by guanine upon formation of double stranded regions has been reported (43). However, the formation of static complexes between Bim and the G in close proximity is still possible inasmuch as the P4 region is only partially paired in the absence of Mg^2+^. The conformational change between ***U*** and ***I*** with the formation of the P4 paired region taking place at [Mg^2+^] ≤10 mM is sufficient to eliminate static quenching. The increase in the residual anisotropy upon the first addition of Mg^2+^ also indicates an increased rigidity of the probe in the intermediate conformation ***I***, reflecting the chelation of Mg^2+^ with the triphosphate on the 5′-end of the **R**-RNA substrate.

### Branching reaction monitored by Bimane

The branching reactions in 40 mM Mg^2+^ and 37°C were carried out at pH 7.5 in order to provide kinetic resolution of the various steps involved. According to Coppins *et al.* (20) the original 7S11 **LER** reaction rate diminishes by ∼90% from pH 9 to 7.5, presumably due to reduction in negative charge at the active site. Javadi-Zarnaghi and Höbartner have reported a *k*_obs_ = 0.013 min^−1^ at pH 7.5 and 40 mM Mg^2+^ for **LER** (21). The Bimane signals permitted a detailed analysis of the steps involved according to a model involving fast (${\textbf{\textit{U}}}{\rightleftharpoons}{\textbf{\textit{I}}}$) and slow (${\textbf{\textit{I}}}{\rightleftharpoons}{\textbf{\textit{N}}}$) conformational transitions with catalysis ensuing from the ***N*** state (Equation (8)). The derived value of *k*_cat_ was ∼0.01 min^−1^ in excellent agreement with the *k*_obs_ cited above. A number of interesting inferences can be derived from the detailed information at 37°C. The ${\textbf{\textit{U}}}{\rightleftharpoons}{\textbf{\textit{I}}}$ transition is less favorable than at 15°C, as would be expected for thermodynamically instable tertiary interactions in the intermediate ***I***. However, the higher temperature greatly favors the active conformation of ***N***. Furthermore, it appears to have a somewhat higher quantum yield (of Bim fluorescence) and, most unexpectedly, a very low steady-state anisotropy ($r_U \;{\rm and}\;r_{I} \simeq 0.05 - 0.05$ compared to $r_N \;{\rm and}\;r_{{\rm Bim - PP_i} } \simeq 0.01).$ The large depolarization cannot be quantitatively accounted by only a slightly (20–30%) longer emission lifetime and thus we conclude that the Bim (triphosphate **R**-terminus) in the ***N*** state is very exposed to the medium at 37°C, with considerable rotational freedom and absence of quenching interactions with the terminal guanine of the **R**-RNA substrate. These features present at 37°C but not at 15°C presumably reflect the active conformation of the 7S11 deoxyribozyme. The Bim-PPi product is still more fluorescent than ***N***, thereby providing an additional distinctive signal as the reaction proceeds. Thus, the first exponential component (*k*_obs1_) reflects primarily the ${\textbf{\textit{I}}}{\rightleftharpoons}{\textbf{\textit{N}}}$ transition as well as some catalysis whereas the second (*k*_obs2_) is dominated by catalysis; these features are apparent in a simulation given in Supplementary Figure S6. The single exponential reaction recorded by the radiolabel based assay ((21), and in this work, Figure 3) only reports the time course of the branched product P_1_, as demonstrated in Supplementary Figure S6. We note, finally, that the continuous ‘on-line’ signals provided by the Bim probe not only are responsive to both conformational changes and branching-cleavage reaction of the deoxyribozyme, but are inherently more convenient and easy to implement than the conventional, necessarily discontinuous assays based on radiolabels.

## SUMMARY

In this study, we have demonstrated that the fluorescence of Bimane can be used to monitor the conformational transitions in a RNA branching deoxyribozyme and the kinetics of the branching reaction it catalyzes. The very small fluorophore can be attached directly to the 5′-triphosphate on the **R**-RNA substrate, allowing labeling the RNA without the necessity for spacers or linkers. The fluorescence parameters reveal that the conformational rearrangements associated with the interaction of the **LER** tri-molecular complex with Mg^2+^ proceed through a two-step mechanism. In the inactive ***U*** conformer and in the absence of Mg^2+^ the overhanging 5′- end bases of the **R**-RNA substrate are mostly unpaired. Addition of Mg^2+^ leads to pairing of these residues and the formation of the P4 helix in the intermediate ***I*** conformation, which are manifested by changes in the emission intensity, steady state anisotropy and time resolved fluorescence. However, the ${\textbf{\textit{U}}}{\rightleftharpoons}{\textbf{\textit{I}}}$ equilibrium is labile and proceeds to the 3HJ of the active ***N*** conformation only upon further addition of Mg^2+^. Attainment of the ***N*** conformation requires the ‘correct’ 7S11 core sequence of the single stranded loops A and B, as demonstrated by the failure of the inactive loop-A mutant **E**(G9A) to undergo step ii in the reaction sequence (Equation (9)). The Bim signals provide continuous monitoring and quantitative characterization of the branching reaction at 37°C. Their analysis yields equilibrium and rate constants, and relative quantum yields and steady-state anisotropies for the various molecular species. For the original 7S11 sequence, at 37°C and 7.5 pH, ***N*** is formed at a rate of 0.2 min^−1^, while the ligation, leading to the branched RNA product and the release of Bim-PP_i_ proceeds at a rate of 0.01 min^−1^. In comparison, the velocities of some naturally occurring ribozymes are 100-fold, in exceptional cases up to 10^5^-fold, greater (44), indicating the need for precise stereochemical positioning for productive nucleophilic attack utilizing efficient reaction ‘strategies’ (45,46). More efficient deoxyribozymes would result from a reduction in self-structure of the individual nucleic acid components (DNA and RNA) and optimization of secondary and tertiary interactions in the presence of specific multivalent cations at temperatures required for catalysis.

## SUPPLEMENTARY DATA

Supplementary Data are available at NAR Online.

SUPPLEMENTARY DATA
