# Supplementary material for: Mg2+-dependent conformational changes and product release during DNA-catalyzed RNA ligation monitored by Bimane fluorescence
Source: Nucleic Acids Res. 2014 Dec 10;43(1):40–50. doi: 10.1093/nar/gku1268 (PMC4288166; doi:10.1093/nar/gku1268)
Supplement: SUPPLEMENTARY DATA [file supp_43_1_40__index.html]

Mg2+-dependent conformational changes and product release during DNA-catalyzed RNA ligation monitored by Bimane fluorescence — SUPPLEMENTARY DATA 

# Mg2+-dependent conformational changes and product release during DNA-catalyzed RNA ligation monitored by Bimane fluorescence

## SUPPLEMENTARY DATA

**Files in this Data Supplement:**

- SUPPLEMENTARY DATA
