# Supplementary material for: Mg2+-dependent conformational changes and product release during DNA-catalyzed RNA ligation monitored by Bimane fluorescence
Source: Nucleic Acids Res. 2014 Dec 10;43(1):40–50. doi: 10.1093/nar/gku1268 (PMC4288166; doi:10.1093/nar/gku1268)
Supplement: SUPPLEMENTARY DATA [file supp_gku1268_nar-01961-f-2014-File010.pdf]

Mg^{2+}-dependent conformational changes and product release during DNA catalyzed RNA ligation monitored by Bimane fluorescence

Elisa Turriani[1,2], Claudia Höbartner[3], and Thomas M. Jovin[2]

[1] Scuola Normale Superiore di Pisa, Piazza dei Cavalieri 7, I-56126 Pisa, Italy

[2] Laboratory for Cellular Dynamics, Max Planck Institute for Biophysical Chemistry, 37077 Göttingen, Germany

[3] Max Planck Nucleic Acid Chemistry Research Group, Max Planck Institute for Biophysical Chemistry, 37077 Göttingen, German

Supporting Material

Oligonucleotide sequences

RNA substrates

L: 5′- GGAUAAUACGACUCAC -3′

L(Ap): 5′- GGAUAAUACGXCUCAC -3′

L(dAp): 5′- GGAUAAUACGdXCUCAC -3′

R(Bim): 5′- YpppGGAAGAGAUGGCGACGG -3′

X = 2Ap, Y = Bim

DNA substrates

E: 5′- CCGTCGCCATCTC**CAGTGCAGGGC**GTGAG**GGCTCGG*TTCC***CGTATTATCC -3′

E(G9A): 5′- CCGTCGCCATCTC**CAGTGCAGAGC**GTGAG**GGCTCGG*TTCC***CGTATTATCC -3′

E(C16G): 5′- CCGTCGCCATCTC**CAGTGCAGGGC**GTGAG**GGCTGGG*TTCC***CGTATTATCC -3′

E(P4): 5′- CCGTCGCCATCTC**CAGTGCAGGGC**GTGAG**GGCTCGG*AAGG***CGTATTATCC -3′

The bold nucleotides in the sequences represent the catalytic loop regions, and the remaining nucleotides constitute the binding regions for RNA substrates. The underlined nucleotides in **E**(G9A), **E**(C16G) and **E**(P4) indicate mutated positions. The bold italic nucleotides belong to loop B of 7S11 and are involved in formation of the paired region P4 with the **R**-RNA substrate

R(Bim) Stability

The stability of the **R**(Bim) product of the labelling procedure towards hydrolysis of the triphosphate was tested by heating the sample at 37 °C for 3 h and recording the fluorescence of the probe at 5 min intervals. Free Bim-PP_i has a higher quantum yield than the unhydrolyzed probe-RNA conjugate; release of the pyrophosphate can thus be detected by the increase in fluorescence. Hydrolysis was detected neither in the absence nor in the presence of 40 mM Mg^{2+}, as evidenced by the constancy of fluorescence over 3 hours, and the reversal by EDTA of the fluorescence increase caused by the addition of 40 mM Mg^{2+} (Supplementary Figure S2).

Kinetic assays

The 5'-^{32}P-labeled left-hand RNA substrate (**L**) was the limiting reagent relative to the deoxyribozyme E and the right-RNA substrate (**R**) (4,13). The **L:E:R** ratio was 1 : 5 : 10 with [**L**] = 0.1 μM. The mixture of **L, E** and **R** was annealed in the presence annealing buffer (5 mM Na-HEPES, pH 7.5, 15 mM NaCl, 0.1 mM EDTA) at 95 °C for 2 min followed by incubation at 25 °C. The reaction was initiated by the addition of 2 μL 5× CHES ligation buffer (containing 250 mM Na-CHES, pH 9.0, 750 mM NaCl, 10 mM KCl) and 1 μL of 400 mM $MgCl_2$ to a final volume of 10 μL. The ligation at 37 °C was followed for 3 h and quenched at appropriate time points by adding a stop solution (80% formamide,1×TBE [89 mM Tris and boric acid, pH 8.3], and 50 mM EDTA containing 0.025% bromophenol blue and xylene cyanol) to aliquots of reaction mixture. The ligated product was separated from the unligated starting material by denaturing PAGE and imaged on a Phosphorimager.

Supplementary Figures and Tables

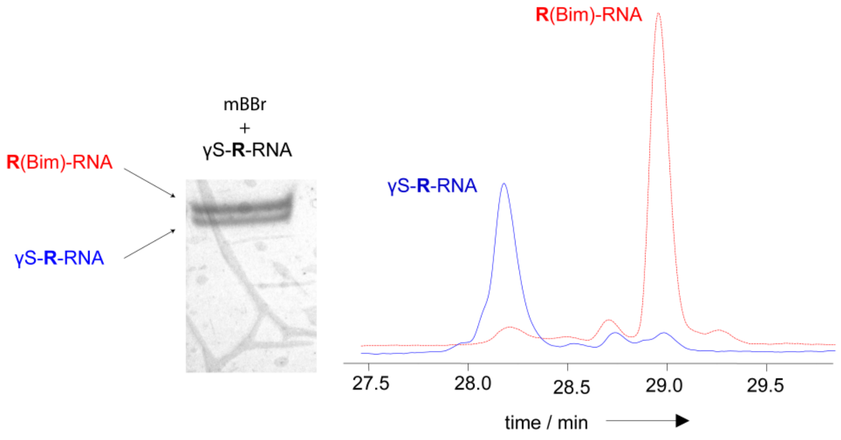

Supplementary Figure S1. Labeling of γ-S-**R**-RNA with mBBr to obtain **R**(Bim)-RNA. The bands of the labeled and un-labeled product on the denaturing PAGE gel are visible under UV light transillumination. The HPLC traces of extracts of the two gel bands indicate the high purity of the labeled product. Anion exchange HPLC, detection at 260 nm.

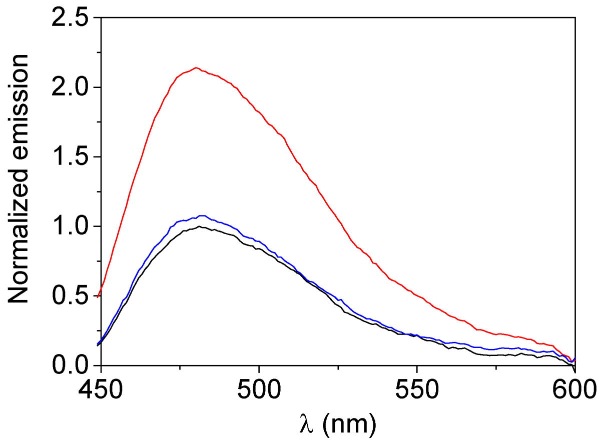

Supplementary Figure S2. Stability of **R**(Bim). Influence of $MgCl_2$ on the fluorescence of Bim in single-stranded **R**(Bim)-RNA at 37 °C. Emission spectra in buffer (black), buffer + 40 mM $MgCl_2$ (red), and buffer + 40 mM $MgCl_2$ + 80 mM EDTA (added after 3 hr, blue). The spectra were normalized to the maximum emission in the absence of $MgCl_2$ (black curve). Conditions: 1 μM **R**(Bim), 150 mM NaCl, 2 mM KCl, 50 mM Na-HEPES, pH 7.5; λ_{exc} = 398 nm.

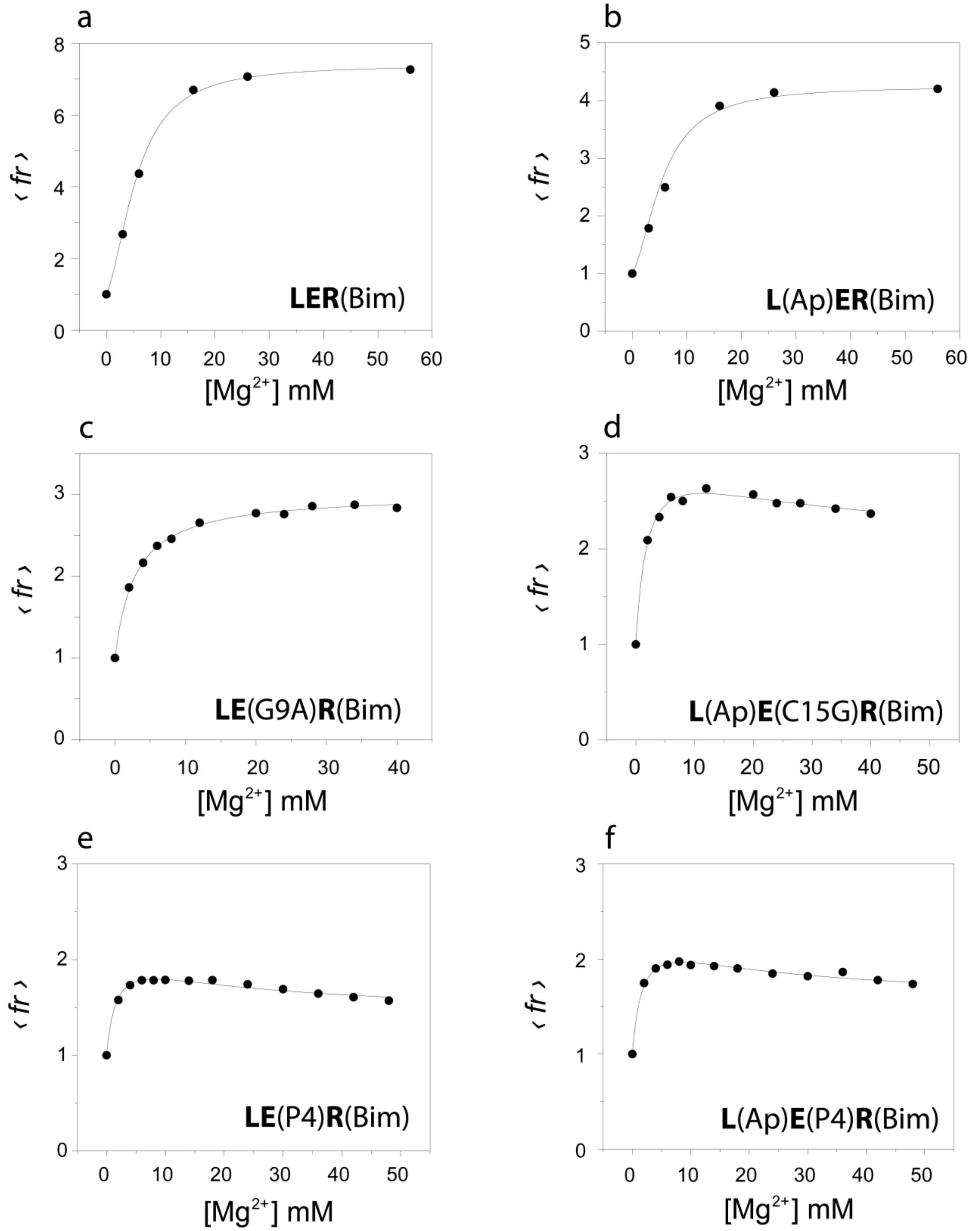

Supplementary Figure S3. Changes in $\langle \mathbf{\mathit{fr}} \rangle$ of the complexes **L**(x)**E**(y)**R**(Bim) upon titration with $MgCl_2$. 1 μM **L**(x)**E**(y)**R**(Bim), 0-56 mM $MgCl_2$, 150 mM NaCl, 2 mM KCl, 50 mM Na-HEPES, pH 7.5; 15 °C; λ_{exc} = 398 nm, λ_{em} = 480 nm. The titrations were analyzed with the two-step equilibrium model (described in Figure 1 and Equation 6), except for **L**(Ap)**E**(G9A)**R**(Bim), which was fit to a single step reaction.

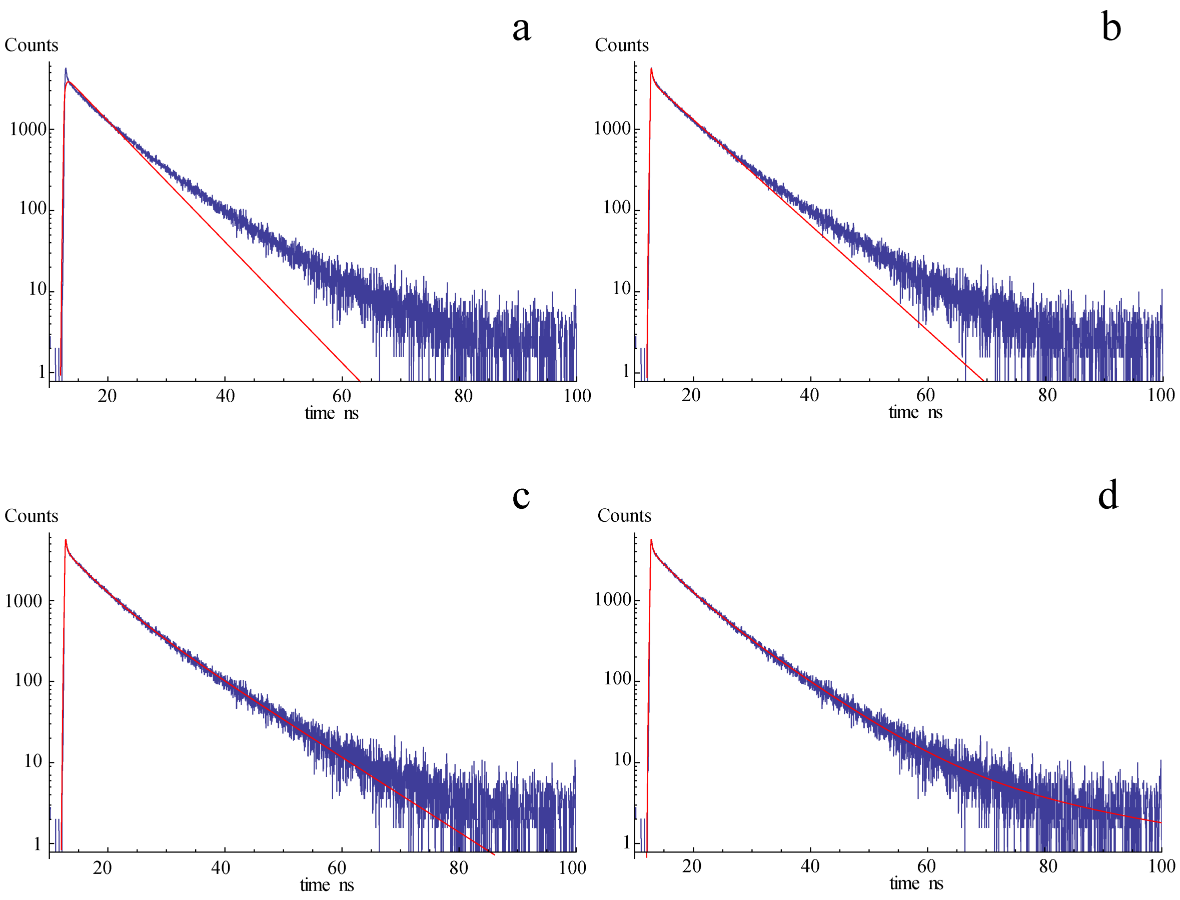

Supplementary Figure S4: Fluorescence decay of **L**(dAP)**ER**(Bim). a-d) fits to 1, 2, 3, or 4 exponential components, respectively**.** Conditions: 4.2 μM **L**(dAp), 4.1 μM **E**, 4.0 μM **R**(Bim).

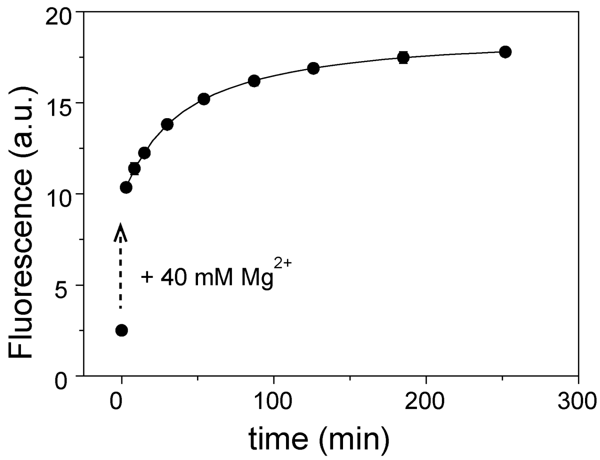

Supplementary Figure S5. Kinetics of branching reaction at 37 °C with Bim labeled **R**-RNA and 2Ap labeled **L**(2Ap)RNA followed by bimane fluorescence. Curve analyzed with a double exponential function: k_{obs1} = 0.046±0.005 min^{-1}, k_{obs2} = 0.011±0.001 min^{-1}. Conditions: 1 μM **L**(Ap)**ER**(Bim), 55 mM $MgCl_2$, 150 mM NaCl, 2 mM KCl, 50 mM Na-HEPES, pH 7.5; 37 °C; λ_{exc} = 398 nm.

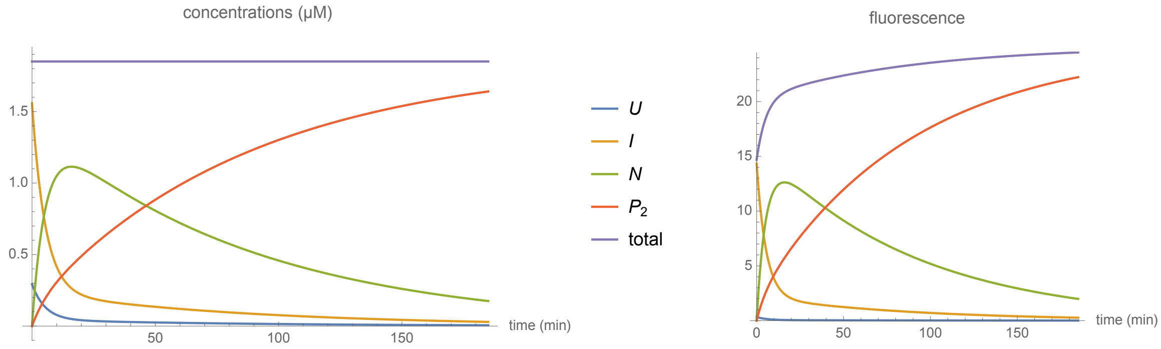

Supplementary Figure S6: Computed time course based on the analysis of the branching reaction of **LER**(Bim) at 37 °C (Figure 8b). The rapid initial transition from ***I*** to ***N*** is apparent in both the concentration and fluorescence curves. The second slower phase (> ~20 min) is dominated by the turnover of ***N***.

Supplementary Table S1: Analysis of the fluorescence decay curves of **L**(dAp)**ER**(Bim) with decay models incorporating 1, 2, 3, or 4 exponentials. No Mg^{2+}.

No. of exponentials	τ_1 ns	τ_2 ns	τ_3 ns	τ_4 ns	τ_{amp} ns	stat[1]
1	5.83±0.03	-	-	-	5.82±0.03	41.0
2	0.068±0.002	6.64±0.01	-	-	0.93±0.02	9.02
3	0.040±0.001	4.3±0.2	9.3±0.3	-	0.59±0.04	2.84
4	0.046±0.002	3.3±0.2	7.7±0.3	22.0±6.0	0.70±0.05	2.95

[1] mean of the squares of the weighted residuals

Supplementary Table S2: [Mg^{2+}] dependence of fluorescence decay parameters of Bim in the trimolecular complex **L**(dAp)**ER**(Bim). Conditions: 4.2 μM **L**(dAp), 4.1 μM **E**, 4.0 μM **R**(Bim).

[Mg^{2+}]	τ_1 ns	τ_2 ns	τ_3 ns	τ_{amp} ns	α_1	α_2	α_3
0	0.040±0.001	4.3±0.2	9.3±0.3	0.59±0.04	0.91±0.01	0.047±0.003	0.038±0.003
2.0	0.043±0.002	3.5±0.1	9.4±0.1	1.04±0.05	0.80±0.04	0.037±0.002	0.093±0.002
5.8	0.056±0.005	4.4±0.2	9.7±0.1	1.55±0.13	0.81±0.07	0.054±0.005	0.132±0.005
9.5	0.054±0.005	4.8±0.2	9.8±0.2	1.56±0.14	0.81±0.08	0.060±0.006	0.126±0.006
16.5	0.047±0.005	4.4±0.2	9.6±0.1	1.42±0.13	0.83 ±0.09	0.048±0.004	0.122±0.004
23.0	0.069±0.005	5.1±0.2	9.9±0.2	1.95±0.16	0.77±0.06	0.085±0.009	0.148±0.009
34.7	0.067±0.005	5.0±0.2	9.8±0.2	1.90±0.16	0.77±0.06	0.080±0.009	0.147±0.009
45.0	0.047±0.005	4.4±0.2	9.6±0.1	1.42±0.14	0.83±0.09	0.052±0.004	0.120±0.004
